# An Interpretable Hybrid SFNet Deep Learning Framework for Multi-Site Bone Fracture Detection in Medical Imaging

**DOI:** 10.3390/diagnostics16070966

**Published:** 2026-03-24

**Authors:** Wijdan S. Aljebreen, Da’ad Albahdal, Shuaa S. Alharbi, Naif S. Alshammari, Haifa F. Alhasson

**Affiliations:** 1 Department of Information Technology, College of Computer, Qassim University, Buraydah 52571, Saudi Arabia; 451214502@qu.edu.sa (W.S.A.); 451214505@qu.edu.sa (D.A.); shuaa.s.alharbi@qu.edu.sa (S.S.A.); 2 Department of Computer Sciences, College of Computer and Information Sciences, Majmaah University, Majmaah 11952, Saudi Arabia; n.alshammari@mu.edu.sa

**Keywords:** artificial intelligence, bone fracture detection, deep learning models, diagnosis, grad-CAM, hybrid SFNet, machine learning, medical imaging, image segmentation

## Abstract

**Background/Objectives**: Accurate bone fracture detection is essential for orthopedic diagnosis and trauma management. Manual interpretation of X-ray or CT images can be time-consuming and may lead to inter-observer variability, particularly in subtle or multi-site fracture cases. This study proposes an interpretable Hybrid Selective Feature Network (Hybrid SFNet) to improve multi-site bone fracture detection performance and boundary localization. **Methods**: The proposed Hybrid SFNet extends the original SFNet architecture by incorporating multi-scale convolutional feature extraction and a semantic flow mechanism to enhance structural representation and fracture boundary delineation. Preprocessing techniques, including Canny edge detection, normalization, and data augmentation, were applied to improve feature quality. Model interpretability was addressed using Gradient-weighted Class Activation Mapping (Grad-CAM) to visualize regions contributing to predictions. The model was evaluated on publicly available multi-site fracture datasets using both standard and class-weighted loss configurations. **Results**: For binary fracture classification, the proposed model achieved 90 accuracy, 94% precision, 77% recall, and an F1-score of 85% for fractured cases. When class-weighted loss was applied, recall improved to 85%, reducing false negatives from 145 to 94 cases (approximately 35%). Under the weighted configuration, Cohen’s Kappa reached 0.79 and the Matthews Correlation Coefficient (MCC) reached 0.76. **Conclusions**: The proposed Hybrid SFNet provides an interpretable and effective framework for multi-site bone fracture detection. The integration of multi-scale feature extraction and semantic flow mechanisms enhances detection performance and boundary localization, while Grad-CAM supports clinical interpretability. These results indicate the model’s potential for supporting clinical decision-making in orthopedic imaging.

## 1. Introduction

The detection and analysis of bone fractures are critical in orthopedic diagnostics and trauma care. While conventional imaging modalities such as X-rays and computed tomography (CT) are widely used, the manual interpretation of these images often results in variability in diagnostic accuracy and delays in treatment. Recent advances in deep learning have demonstrated AUROC values ranging from 0.929 to 0.98 in fracture detection tasks [1]. However, detection of multi-site fractures with their distinctive diagnostic challenges has attracted relatively less interest in the literature. Within this work, multi-site fracture detection refers to building a single unified deep learning model capable of detecting fractures from multiple sites in the body (wrist, femur, rib, etc.) as opposed to detecting multiple fractures within one image. This distinction affects model generalization, as heterogeneous anatomical regions exhibit different imaging characteristics.

Semantic segmentation-based models have emerged as a robust approach for medical image analysis. Among these, the SFNet (Semantic Flow Network) architecture has demonstrated state-of-the-art performance in applications requiring pixel-wise predictions [2]. Although SFNet has demonstrated strong performance in dense prediction tasks such as optical flow estimation, its direct application to multi-site medical image classification requires architectural adaptation and domain-specific enhancements. Hybrid models that combine feature extraction with hierarchical contextual understanding present a promising direction to address these challenges.

The recent work, such as SPX-GNN [3], developed an explainable graph neural network to model long-range dependencies in chest X-ray tuberculosis classification. By incorporating graph-based structural reasoning, the model enhances contextual understanding and interpretability in radiographic analysis. However, such graph-based modeling strategies have not yet been extensively explored for multi-site fracture detection tasks, particularly within unified CNN-based frameworks.

Despite advancements in deep learning for medical imaging, several challenges remain. The detection of multi-site fractures is an understudied area, and existing state-of-the-art models, often designed for specific bones (e.g., femur or tibia), lack generalizability across different anatomical regions. Furthermore, small datasets and noisy data, such as minor or low-contrast fractures, reduce model accuracy and reliability. Data augmentation techniques and methods to handle noisy inputs are underutilized in this domain. Additionally, deploying deep learning models in clinical settings remains difficult due to issues such as interpretability, computational efficiency, and integration with existing workflows. Moreover, current systems rarely combine imaging data with structured clinical metadata (e.g., patient age, trauma mechanism, or radiographic projection). While multimodal integration has great potential for enhancing diagnostic performance, the majority of fracture datasets available to the general public do not offer standardized clinical metadata, making such practices infeasible.

This research addresses these challenges by introducing a Hybrid SFNet model specifically designed for multi-site fracture detection. The proposed model leverages convolutional layers and semantic flow mechanisms to enhance feature localization and boundary identification. It incorporates custom layers and a hierarchical feature extraction strategy to achieve robust detection performance, even in cases of low-contrast fractures or overlapping anatomical structures. The objectives of this study include providing probabilistic outputs for fractures to aid clinical decision-making, generating heatmaps for fracture localization to improve visualization, and enhancing model interpretability using Grad-CAM (Gradient-weighted Class Activation Mapping). By addressing these gaps, the Hybrid SFNet model aims to achieve balanced performance across accuracy, recall, and MCC while maintaining approximately 110,785 trainable parameters.

The primary novelty of this work lies in the development of a unified multi-site fracture detection framework capable of generalizing across heterogeneous anatomical regions within a single model, in contrast to most existing approaches that are designed for specific bones or isolated anatomical sites. This research proposes a hybrid adaptation of the Semantic Flow Network (SFNet) that was initially developed for optical flow estimation and adapts the network for the medical classification of fractures by performing multi-scale feature fusion and semantic flow–guided boundary refinement. Furthermore, the framework supports edge-enhanced dual-channel input with Canny edge detection, an imbalance-aware optimization with class-weighted loss to enhance fracture sensitivity, and Grad-CAM-based interpretability to promote clarity in clinical decision-making. Taken together, these contribute to an anatomically generalized, sensitivity-oriented, and explainable deep learning framework for multi-site bone fracture detection.

## 2. Summary of Bone Fracture Detection Techniques

Deep learning techniques have emerged as a transformative tool for bone fracture detection, leveraging methods such as Convolutional Neural Networks (CNNs), transfer learning, object detection models, and image segmentation. This section provides a comprehensive summary of the key contributions in these areas.

### 2.1. Convolutional Neural Networks (CNNs) and Transfer Learning

CNNs and transfer learning have been widely applied in bone fracture detection, offering robust feature extraction and classification capabilities. Sinthura et al. [4] proposed a system that utilized Discrete Wavelet Transform (DWT) for edge detection and Spatial Fuzzy C-Means (SFCM) clustering. Their model achieved an accuracy of 78%, but they noted the need to address noise in X-ray images in future work. Wang et al. [5] employed a U-Net for segmentation and ResNet-50 for fracture classification using a dataset of 686 mandibular fractures. Their model achieved a Dice coefficient of 0.943, an average AUC of 0.965, and the highest accuracy of 98.28% in specific subregions. They emphasized the need for diverse datasets, particularly for alveolar process fractures.

Kim et al. [6] explored transfer learning with Inception v3 on 1389 lateral wrist radiographs. Their model achieved an AUC of 0.954, a sensitivity of 0.9, and a specificity of 0.88. However, they observed slight overfitting in their results. Similarly, Ma et al. [7] developed a two-stage system that combined Faster R-CNN for bone localization and CrackNet for fracture detection, with Schmid filters enhancing fracture line detection. Their model achieved an accuracy of 90.11%, a precision of 89.73%, a recall of 90.49%, and an F-measure of 90.14%. They suggested that future research focus on one-stage systems for simultaneous localization and detection. Lindsey et al. [8] extended the U-Net by integrating probabilistic fracture detection and heatmap generation, achieving an AUC of 0.967 for wrist fractures. Guy et al. [9] compared AlexNet and GoogLeNet for anterior–posterior pelvic fracture detection, with GoogLeNet achieving an accuracy of 90.9% and an AUC of 0.98, outperforming AlexNet’s accuracy of 85.5% and AUC of 0.95. Their results indicated that radiologists (93.5% accuracy) and radiology residents (92.9% accuracy) outperformed both models. Rajpurkar et al. [10] trained a 169-layer DenseNet on the MURA dataset, consisting of 40,561 musculoskeletal radiographs. Their model achieved an AUROC of 0.929, a sensitivity of 81.5%, and a specificity of 88.7%. However, the model underperformed compared to radiologists in certain fracture types, such as humerus and forearm fractures.

Transfer learning has demonstrated its potential to enhance fracture detection accuracy by leveraging pre-trained models. Baig et al. [11] applied AlexNet with transfer learning on a Kaggle dataset of 8863 X-rays. Their model achieved an accuracy of 98.43%, with a precision, recall, and F1-score of approximately 0.982 for fracture detection. Similarly, Amodeo et al. [12] used ResNet-50, pre-trained on ImageNet, and fine-tuned it on a dataset of 208 CT scans. Their model achieved an accuracy of 81%, a recall of 51%, a precision of 50%, and an AUC of 0.82. They emphasized the need for improving small fracture detection in future studies.

Haitaamar et al. [13] applied the U-Net for rib fracture detection and semantic segmentation, achieving an accuracy of 95%. Nguyen et al. [14] used YOLOv4 for arm bone fracture detection, achieving an accuracy of 81.91%, highlighting the effectiveness of object detection models for localizing fractures. Abbas et al. [15] utilized Faster R-CNN for tibia–fibula bone fracture detection, achieving an accuracy of 97%, emphasizing its utility in long bone fractures. Sasidhar et al. [16] experimented with transfer learning using VGG19, DenseNet121, and DenseNet169 architectures, achieving an accuracy of 92% for humerus bone fracture detection.

Moreover, recent papers improved underlying architecture designs of deep learning–based fracture detection systems and their clinical applications. Aldhyani et al. [17] proposed a deep learning framework for automated diagnosis of bone fractures in radiographic images and proved CNN-based classification approaches to be effective systems for the clinical routine. Similarly, Alwzwazy et al. [18] proposed *FracNet*, an end-to-end deep learning architecture for detecting bone fractures, focused on efficient feature extraction and inference. Yu et al. [19] used deep learning integrated with bone morphometric parameter input to improve fracture analysis for early prediction of fracture non-union, emphasizing the shift from imaging features with structural model descriptions. More recently, Belali et al. [20] developed a deep learning–based X-ray analysis framework for osteoporosis-associated fracture detection, emphasizing enhanced sensitivity to subtle fracture features. Architectural comparisons have also been investigated. For example, Salman and Abu-Naser [21] compared MobileNetV2 and ResNet50 for fracture detection, reporting performance differences associated with backbone selection. Furthermore, Bhat et al. [22] was an exploratory study of a fracture identification tool for hand radiographs based on deep machine learning and traditional machine learning, which highlights that further progress is needed in hybrid and domain-specific methods. Although these works show some development on automated fracture analyses, the majority of their methodologies fall under single anatomical/backbone-specific comparisons. A novel method in the Hybrid SFNet framework aims to deliver a single multi-site fracture detection with enhanced feature fusion and interpretability.

By leveraging CNNs and transfer learning, researchers have demonstrated significant improvements in fracture detection and classification. However, challenges such as small dataset sizes, overfitting, and limited generalizability remain. Table 1 summarizes studies employing Convolutional Neural Networks (CNNs) and transfer learning for bone fracture detection.

### 2.2. Object Detection Models and Image Segmentation

Object detection models and image segmentation techniques have been pivotal in automating bone fracture detection. Object detection models, such as YOLO and Faster R-CNN, have been adapted to simultaneously localize and classify fractures. Moon et al. [23] used the YOLOX-S model with IoU loss and Mixup data augmentation on private CT images, achieving an average precision of 69.8%. Hardalacc et al. [24] applied Faster R-CNN, RetinaNet, and ensemble models to a dataset of 542 wrist X-rays. Their best-performing ensemble model, WFD-C, achieved an AP50 score of 0.8639 and an optimal localization recall-precision (LRP) score of 0.77. However, the lack of healthy controls in their study limited the model’s generalizability.

Shan et al. [25] combined YOLOv3 for object detection with a modified attention U-Net for segmentation. Their YOLOv3 model achieved a precision of 0.894, a recall of 0.587, and an accuracy of 83.83%, while the segmentation model achieved an accuracy of 88.26%. Similarly, Hrvzic et al. [26] applied a modified U-Net and YOLOv4 to the publicly available GRAZPEDWRI-DX dataset, consisting of 20,327 wrist X-rays. Their best-performing model achieved a precision of 0.90, a recall of 0.89, an F1-score of 0.89, and an accuracy of 0.89. Ju et al. [27] extended this work using YOLOv8, achieving a mean average precision (mAP50) of 0.638 and detecting nine trauma classes.

Image segmentation techniques have been instrumental in improving fracture detection by isolating fractures from surrounding structures. Wang et al. [28] proposed ParallelNet, a multiple backbone network for thigh bone fracture detection, achieving an accuracy of 87.8%. Wang et al. [29] introduced a DCNN with attention mechanisms for multi-site fracture detection, achieving an accuracy of 88.7%. Hrvzic et al. [30] proposed a hybrid method combining local Shannon entropy and PCA for pixel-level segmentation and edge detection, achieving an accuracy of 91.16%.

Despite advancements, challenges such as small datasets, subtle fractures, and limited generalizability persist. Future research should explore integrating these techniques into clinical workflows to improve diagnostic accuracy and efficiency. Table 2 summarizes studies that utilize object detection models and image segmentation techniques for bone fracture detection.

## 3. Materials and Methods

**Figure 1:**
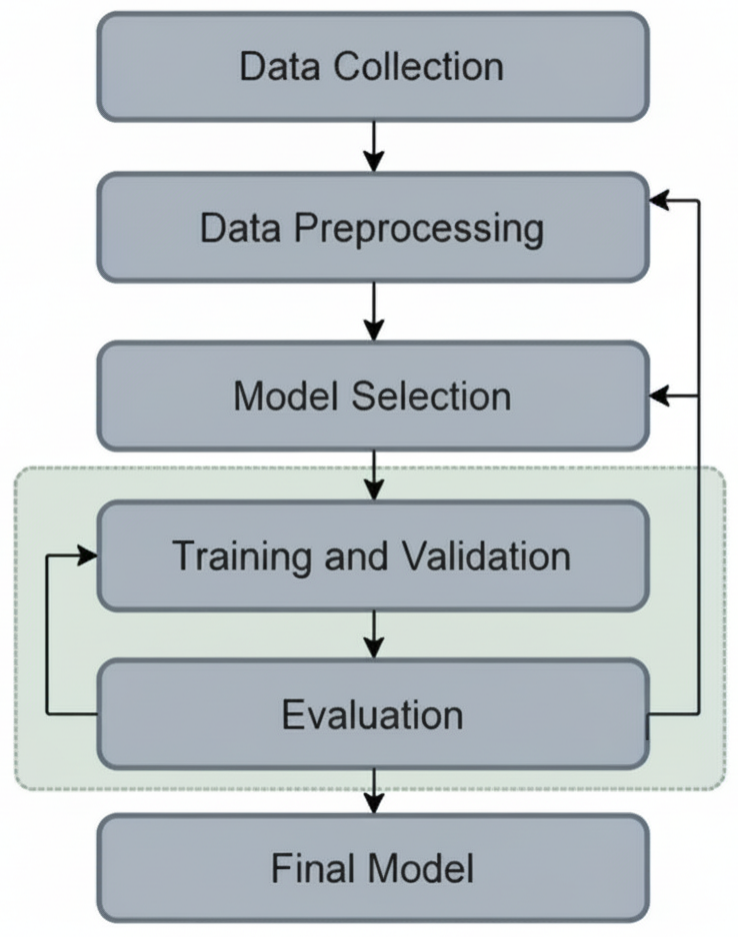
Overview of the proposed research methodology.

This section outlines the methodology for detecting multi-site bone fractures using the proposed Hybrid SFNet deep learning model. The research involves designing, developing, and evaluating the model and performing comparative analyses against other state-of-the-art models. The methodology aims to optimize classification accuracy and reduce computational complexity (110,785 trainable parameters) of multi-site bone fracture detection. Figure 1 illustrates an overview of the research methodology.

### 3.1. Data Collection

**Figure 2:**
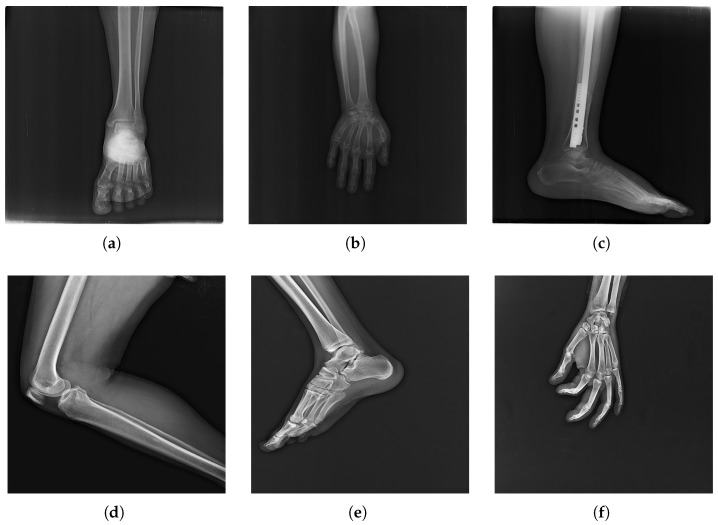
Sample of X-ray images from *FracAtlas* [31], where (**a**–**c**) showcase fractures and (**d**–**f**) do not contain fractures.

The datasets used in this study are the publicly available FracAtlas [31] and Diagnostic Imaging Dataset (DIDS) [32]. The FracAtlas dataset contains 4083 X-ray images, with 3605 fractured cases and 808 non-fractured cases, representing a significant class imbalance. The fractures include wrist (35%), femur (25%), and rib fractures (15%). Figure 2 presents examples of X-ray images from *FracAtlas*. The DIDS dataset complements this by providing 717 fractured and 3366 non-fractured cases, resulting in a more balanced class distribution. Both datasets primarily comprise X-ray images with resolutions of 512 × 512 px (FracAtlas) and 1024 × 1024 px (DIDS).

The FracAtlas dataset was annotated by two radiologists and one orthopedic expert to ensure high-quality labeling. Annotations include fracture classification, localization, and segmentation masks provided in multiple formats, such as COCO, VGG, YOLO, and Pascal VOC. The DIDS dataset is sourced from public repositories that provide labeled fracture data suitable for deep learning tasks.

Both datasets predominantly include adult patients aged 18–65 years, with a roughly equal gender distribution in FracAtlas. Table 3 summarizes the key characteristics of these datasets.

To address the class imbalance issue in the first dataset, a second dataset was incorporated to train the model. The Diagnostic Imaging Dataset (DIDS) [33] is a publicly available dataset containing 717 X-ray images with fractures and 3366 images without fractures, resulting in a more balanced class distribution. This balanced dataset is crucial for mitigating the risk of class bias and improving fracture recall. across both fractured and non-fractured cases. Moreover, the DIDS dataset encompasses a variety of anatomical regions, further diversifying the training samples and strengthening the model’s ability to generalize to different types of fractures. The combination of FracAtlas and DIDS increases the size of the training dataset and introduces complementary feature distributions, supporting model training across diverse X-ray imaging conditions. This integration is essential for building a robust and reliable system for fracture detection and classification. Table 4 shows the distribution of the combined dataset after merging the two original datasets. The data is divided into three subsets: the training set (64% of the data), the validation set (16%), and the test set (20%). Each subset includes the number of fractured and non-fractured images, along with the total number of samples.

### 3.2. Data Preprocessing

Preprocessing is essential for enhancing input data quality and ensuring the model can learn relevant features. For X-ray images, the process involves converting them to grayscale, applying the Canny edge filter to highlight boundaries, resizing the images for uniformity, and concatenating features along the channel dimension. These steps prepare the data for effective model training.

#### 3.2.1. Grayscale Conversion

RGB images are converted to grayscale to reduce input dimensionality, simplifying processing while focusing on the structural features of the image. This step helps eliminate redundant color information, allowing the model to concentrate on intensity variations that are critical for analyzing X-ray images.

#### 3.2.2. Canny Edge Detection

The grayscale images are processed using the Canny edge detection algorithm to emphasize edges and contours, highlighting key features such as object boundaries. Canny edge detection involves several steps, including calculating the gradient magnitude and gradient direction, which are defined as follows:

(1) $$M\left(x,y\right)=\sqrt{{\left(G_{x}\left(x,y\right)\right)}^{2}+{\left(G_{y}\left(x,y\right)\right)}^{2}}$$

(2) $$\theta \left(x,y\right)=\tan^{-1}\left(\frac{G_{y}\left(x,y\right)}{G_{x}\left(x,y\right)}\right)$$

where $G_{x}\left(x,y\right)$ and $G_{y}\left(x,y\right)$ represent the gradients of the image in the *x* and *y* directions, respectively.

**Figure 3:**
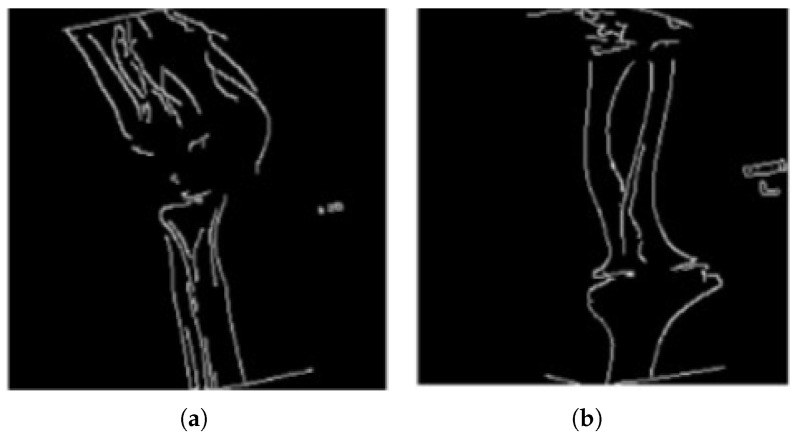
Sample X-ray images processed with Canny edge detection: (**a**) shows a fractured bone, while (**b**) shows a normal bone with no fracture.

Canny edge detection applies non-maximum suppression and double thresholding to refine the edges. Non-maximum suppression thins edges to local maxima along the gradient direction, ensuring only the most prominent edges are retained. Double thresholding is then used to classify edges as strong or weak based on high and low threshold values, enabling the identification of significant features. Figure 3 presents samples of X-ray images after applying Canny edge detection.

#### 3.2.3. Image Reshaping and Concatenation

Both the grayscale and edge-detected images are reshaped into three-dimensional tensors and concatenated along the channel dimension. This process creates a two-channel input for the neural network, combining intensity and edge information to enhance feature representation for model training.

#### 3.2.4. Normalization

The pixel values of the combined image are normalized to a range between 0 and 1. Normalization ensures that all features contribute equally during training and prevents larger values from dominating the learning process. For an image with pixel values I(x,y), the normalization formula is defined as

(3) $$I_{\mathrm{norm}}\left(x,y\right)=\frac{I(x,y)-\mathrm{Min}}{\mathrm{Max}-\mathrm{Min}},$$

where I(x,y) is the original pixel value, and Min and Max are the minimum and maximum pixel values in the image, respectively.

#### 3.2.5. Augmentation

To improve model generalization of the underlying models and to balance imbalanced class data, data augmentation was applied only on the training set. Augmentation was carried out via Keras ImageDataGenerator and applied to the data in the training set online. The following transformations happened randomly for each image at each epoch: rotation of ±15°, flipping in the horizontal direction with a probability of 0.5, random zoom (a random value between 0.9 and 1.1), width and height shifts of 10% of the image or less, and a shift in the brightness of 0.8–1.2. These transformations simulate variations in orientation, scale, position, and illumination while preserving fracture-related image features. No augmentation was applied to the validation or test sets to ensure fair evaluation of performance. Moreover, this augmentation added to the effective training dataset from 5748 images to 120,307 samples with a 12.75-fold increase, which improved the robustness of the model and reduced overfitting by providing the same sample with diverse examples of fractured cases as well as non-fractured cases.

**Figure 4:**
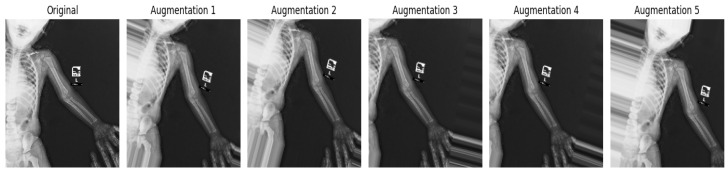
Sample of the augmentation images.

After the augmentation process, the dataset expanded to 42,007 fractured images and 78,300 non-fractured images, totaling 120,307 samples. Figure 4 shows an example of an original image along with its augmentations. Table 5 summarizes the number of images before and after augmentation for each class. This augmentation step increased the size of the training dataset and exposed the model to varied representations of fracture and non-fracture images during training.

This streamlined preprocessing pipeline enhances the model’s capacity to effectively learn both global and local features from the X-ray images. By ensuring that the input data is consistently prepared and augmented, the model is exposed to a diverse range of representations, helping it capture subtle patterns and variations in fracture characteristics. This process reduces noise and irrelevant image information prior to feature extraction during model training. The enhanced preprocessing further aids in mitigating overfitting by introducing variability, enabling the model to generalize better to unseen data.

### 3.3. Proposed Hybrid SFNet

The Hybrid Selective Feature Network (SFNet), a multi-scale feature fusion model, was chosen for this research due to its exceptional ability to balance computational efficiency and high accuracy, particularly in the domain of medical imaging [32]. With approximately 5 million parameters, SFNet is engineered to optimize computation time per epoch while maintaining robust performance. This efficiency makes it highly suitable for applications where both speed and precision are critical, such as medical diagnostics. By achieving this balance, SFNet addresses the practical demands of real-world scenarios, where timely and accurate diagnoses are required in time-sensitive clinical environments. Its design ensures that computational constraints do not compromise diagnostic reliability, ultimately supporting better patient outcomes and advancing the adoption of AI in healthcare.

#### 3.3.1. Model Architecture

The Hybrid SFNet architecture is composed of two distinct feature extraction modules, each consisting of six convolutional layers. These layers are designed with progressively increasing filter sizes—16, 32, 64, 128, 256, and 512—to capture a wide range of spatial features from the input X-ray images. This progressive scaling ensures that the model can effectively learn both fine-grained local features and broader global patterns. After each convolutional operation, the ReLU activation function is applied to introduce non-linearity, followed by batch normalization to stabilize and accelerate the learning process.

Additionally, global average pooling is utilized to reduce the dimensionality of the extracted features while retaining their most important spatial information. This reduces the number of processed features and restricts the model to a subset of high-activation feature maps during training. These design elements collectively contribute to a more robust and practical architecture for medical image analysis.

**Figure 5:**
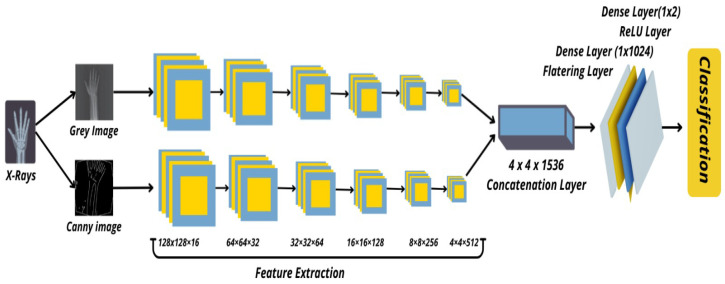
Architecture of Hybrid SFNet Model.

The overall architecture of the Hybrid SFNet model is depicted in Figure 5, which provides a visual overview of its components and their interactions.

The detailed layer configuration of the proposed SFNet is summarized in Table 6, and the training hyperparameters are presented in Table 7.

#### 3.3.2. Feature Fusion and Classification

To integrate information from multiple scales, the outputs from both feature extraction modules are concatenated into a single, comprehensive feature tensor. This fusion mechanism allows the model to combine fine and coarse-grained details from the input images, resulting in a richer representation of the data. By capturing these multi-scale features, the Hybrid SFNet enhances its ability to identify subtle fracture patterns as well as broader structural abnormalities, which is remains necessary to reduce the 23% false-negative rate. The fused feature tensor undergoes flattening and is passed through a dense layer containing 1024 neurons, where the extracted features are further processed and refined. This dense layer is followed by a ReLU activation function to introduce non-linearity and ensure the model captures complex relationships between features. To prevent overfitting, dropout layers are added, which randomly deactivate a fraction of neurons during training to improve generalization.

Finally, the model performs binary classification through a dense output layer with two neurons, corresponding to the classes of “healthy” and “fractured.” The Softmax activation function is applied to convert the raw logits into probability values, ensuring precise classification outcomes. This end-to-end design enables the Hybrid SFNet to deliver both efficient and accurate predictions, making it well-suited for clinical applications.

#### 3.3.3. Advantages and Implications

The Hybrid SFNet’s multi-scale feature fusion approach, combined with its lightweight architecture, ensures that it can process large volumes of medical images efficiently without sacrificing diagnostic accuracy. The use of advanced techniques such as batch normalization, global average pooling, and dropout layers enhances the model’s robustness and generalization capabilities. These attributes make the Hybrid SFNet particularly well-suited for real-world deployment in medical imaging workflows, where reliability, speed, and precision are paramount.

### 3.4. Model Development

For this study, a deep learning model was developed for fracture detection using the Fracatlas dataset [31]. The selected architecture was an SFNet, a complex yet efficient neural network designed to capture critical features from medical images through specialized layers. The dataset was divided into training, validation, and testing sets to ensure the model learned generalizable patterns while minimizing the risk of overfitting.

The model is trained for 20 epochs, with early stopping employed to optimize computational efficiency and prevent overfitting. Training is halted if validation accuracy fails to improve for five consecutive epochs. This strategy ensured a balance between performance and computational cost. The architecture relied on convolutional and max-pooling layers to effectively extract spatial features from the X-ray images, while dense layers transformed these features into representations suitable for classification. Dropout layers are incorporated to reduce overfitting by randomly deactivating neurons during training, promoting better generalization.

To further evaluate the model, Gradient-weighted Class Activation Mapping (Grad-CAM) was employed. This interpretability technique generated heatmaps that highlighted regions of the image most influential to the model’s predictions. These visualizations confirmed that the model primarily focused on medically relevant areas, such as fracture lines or critical edges, validating its decision-making process.

Overall, the final model leveraged careful parameter tuning, effective data handling, Canny edge preprocessing, and robust regularization techniques. These elements contributed to an efficient and reliable tool for automated fracture detection in X-ray imaging, demonstrating its potential for real-world clinical applications.

### 3.5. Evaluation Matrix Used to Evaluate Model

#### 3.5.1. Class-Weighted Binary Cross-Entropy Loss

To address the remaining class imbalance between fractured and non-fractured cases, a class-weighted binary cross-entropy (BCE) loss function was implemented. In this formulation, a larger weight is assigned to the minority (fractured) class, increasing its contribution to the total loss during optimization. The weighted loss function is defined as follows:

(4) $$L=-\frac{1}{N}\sum_{i=1}^{N}\left[w_{1}y_{i}\log\left({\hat{y}}_{i}\right)+w_{0}\left(1-y_{i}\right)\log\left(1-{\hat{y}}_{i}\right)\right]$$

where:

- $y_{i}\in \left\{0,1\right\}$ represents the ground-truth label;
- ${\hat{y}}_{i}$ denotes the predicted probability;
- $w_{1}$ and $w_{0}$ correspond to the weights assigned to fractured and non-fractured classes, respectively.

The class weights were computed inversely proportional to class frequencies in the training set, such that fractured samples receive a higher priority during optimization.

#### 3.5.2. Confusion Matrix and Metrics

The confusion matrix is a vital tool for evaluating the performance of the model. It provides a detailed breakdown of the model’s predictions, offering insights into its strengths and weaknesses. The matrix consists of the following components:

- True Positives (TPs): instances where fracture presence was correctly predicted by the model.
- False Positives (FPs): incorrect predictions where the model identified a fractured bone, but the actual label was healthy.
- True Negatives (TNs): instances where the absence of fracture was correctly predicted by the model.
- False Negatives (FNs): incorrect predictions where the model identified a healthy bone, but the actual label was fractured.

Multiple evaluation metrics are calculated to comprehensively assess model performance:

- Accuracy: the proportion of correct predictions to the total number of samples:
  (5) $$\mathrm{Accuracy}=\frac{\mathrm{TP}+\mathrm{TN}}{\mathrm{TP}+\mathrm{FP}+\mathrm{TN}+\mathrm{FN}}$$
- Precision: the proportion of correctly identified fractured bones to all samples predicted as fractured:
  (6) $$\mathrm{Precision}=\frac{\mathrm{TP}}{\mathrm{TP}+\mathrm{FP}}$$
- Recall (Sensitivity): the proportion of correctly identified fractured bones to all actual fractured bones:
  (7) $$\mathrm{Recall}=\frac{\mathrm{TP}}{\mathrm{TP}+\mathrm{FN}}$$
- F1-Score: the harmonic mean of precision and recall, balancing their contributions:
  (8) $$F1\text{-}\mathrm{Score}=2\;\cdot \;\frac{\mathrm{Precision}\;\cdot \;\mathrm{Recall}}{\mathrm{Precision}+\mathrm{Recall}}$$

These metrics provide a well-rounded evaluation of the model, highlighting its ability to identify fractures effectively while minimizing false positives and false negatives.

#### 3.5.3. Cohen’s Kappa

Cohen’s Kappa indicates the degree of agreement between predicted labels and true labels while accounting for agreement occurring by chance. It is defined as

(9) $$\kappa =\frac{p_{o}-p_{e}}{1-p_{e}}$$

where $p_{o}$ represents the observed agreement (accuracy), and $p_{e}$ denotes the expected agreement by random chance.

The interpretation of κ values is commonly categorized as follows:

- κ<0.40: poor agreement;
- 0.40≤κ<0.60: moderate agreement;
- 0.60≤κ<0.80: substantial agreement;
- κ≥0.80: almost perfect agreement.

Kappa punishes imbalanced predictions versus accuracy and yields a more realistic estimate of classification reliability in clinical scenarios. The Matthews Correlation Coefficient is one of the most trusted single-value performance metrics used to analyze the performance of binary classification, where data is often imbalanced.

#### 3.5.4. Matthews Correlation Coefficient (MCC)

The Matthews Correlation Coefficient (MCC) integrates all four elements of the confusion matrix (TP, TN, FP, FN) and is formulated as

(10) $$MCC=\frac{TP\times TN-FP\times FN}{\sqrt{\left(TP+FP)(TP+FN)(TN+FP)(TN+FN\right)}}$$

MCC values range from:

−1: complete disagreement;
- 0: random prediction;
- +1: perfect prediction.

MCC is very well suited to safety-critical medical AI activities, such as the detection of fractures, in that it balances sensitivity and specificity while accounting for class distributions.

#### 3.5.5. Clinical Relevance

The inclusion of κ and MCC provides additional assessment beyond overall accuracy by incorporating chance-corrected agreement and performance under class imbalance. This is especially important in fracture detection, where reducing false negatives is clinically more critical than maximizing overall accuracy. By incorporating these additional metrics, the proposed Hybrid SFNet framework is evaluated under a more rigorous and clinically meaningful performance framework.

#### 3.5.6. Training and Overfitting Prevention

To ensure the model generalizes well and does not overfit the training data, multiple strategies are employed:

- Early stopping: training is halted if the validation loss does not improve for a predefined number of epochs, preventing overfitting.
- Dropout layers: randomly deactivating neurons during training encourages the model to learn more robust features.
- Batch normalization: normalizing the inputs to each layer during training stabilizes learning and improves convergence speed.

The model’s loss is monitored across training epochs to ensure convergence and stability. These regularization techniques ensure the model maintains high performance on unseen data.

This evaluation methodology combines loss minimization and overfitting prevention techniques to ensure the development of a reliable and effective deep learning model for multi-site bone fracture detection. By optimizing accuracy, efficiency, and robustness, the model is well-suited for real-world applications, offering timely and accurate diagnoses in complex X-ray imaging scenarios where patient outcomes are critical. In addition, the difference between final training accuracy (95%) and validation accuracy (90%) remained below 6%, which is generally considered acceptable in deep learning–based medical imaging studies.

#### 3.5.7. Grad-CAM Computation Process

Gradient-weighted Class Activation Mapping (Grad-CAM) calculates the gradients of the target class score (e.g., fractured or non-fractured) with reference to the feature maps of the last convolutional layer. These gradients are then scaled by their global average to highlight the most important areas in the input image that led to the decision. The heatmap is obtained using a ReLU function in order to consider only positive contributions, which is easier to understand in the model’s attention. X-ray images may be processed to identify fracture-relevant portions of the images, facilitating validation of fracture detection models and clinical decision support.

## 4. Results and Discussion

After implementing the Hybrid SFNet model on the bone fracture dataset, its performance is evaluated to assess its effectiveness in detecting multi-site fractures. The results demonstrate that the model achieved 90% accuracy with 94% precision and 77% recall for fractured cases. Validation accuracy plateaus around 90% after the fourth epoch, with small oscillations between epochs 5–8. This behavior demonstrates mild overfitting tendencies, but the difference between training and validation accuracy was around 5–6%, suggesting that such generalization was controlled. The classification report revealed an overall accuracy of 90%, with precision and recall for Class 0 (non-fracture sites) at 89% and 98%, respectively, resulting in an F1-score of 93%. For Class 1 (fracture sites), precision was 94%, recall was 77%, and the F1-score was 85%. The macro-averaged metrics were 92% for precision, 87% for recall, and 90% for the F1-score, while weighted averages aligned closely, reflecting balanced performance across both classes.

The confusion matrix further highlighted the model’s effectiveness: out of 1171 samples in Class 0, 1142 were correctly classified, with only 29 misclassified, while for Class 1, 481 out of 626 samples were accurately detected, with 145 misclassifications. These results underline the Hybrid SFNet model’s correctly classified 1142/1171 non-fractured cases and 481/626 fractured cases.

Although the model achieved high overall accuracy (90%) and precision (94%) for fractured cases, the recall of 77% for fractures suggests approximately 23% of fracture cases were misclassified as non-fractured. Clinically, in trauma radiology, there is the potential for false negatives to delay diagnosis, inappropriate management, prolonged patient discomfort, or complications. However, in real-world emergency and orthopedic settings, sensitivity (recall) for fractures is frequently emphasized over overall accuracy to decrease missed injuries. Consequently, the proposed Hybrid SFNet achieves 90% accuracy and 0.78 Cohen’s Kappa.

**Figure 6:**
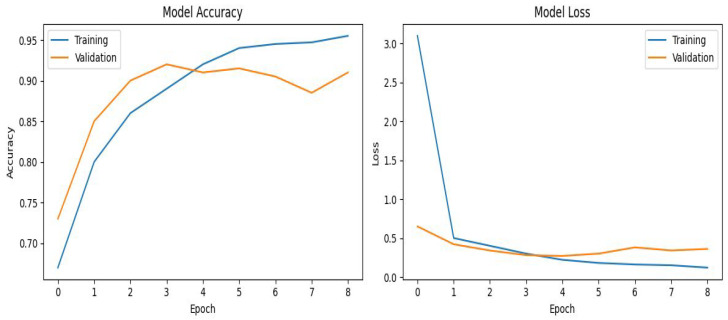
Training and validation loss and accuracy.

Figure 6 illustrates the training and validation loss and accuracy curves for the Hybrid SFNet model. These curves provide a clear visualization of the model’s performance over successive epochs, highlighting the steady improvement in training accuracy, the stabilization of validation accuracy, and the consistent reduction in both training and validation loss. Table 8 summarizes the performance metrics of the Hybrid SFNet model, including precision, recall, and F1-score, for both non-fractured and fractured cases, along with the overall accuracy of the model.

Although small differences in both training and validation curves are detectable after the fourth epoch, early stopping was used to avoid excessive overfitting. The validation loss did not develop a continuous upward trend, and the performance metrics on the independent test set remained stable (accuracy = 0.90). This reflects that minor fluctuations exist but that the model does not exhibit severe overfitting.

### 4.1. Impact of Class-Weighted Loss on Fracture Sensitivity

To further improve fracture sensitivity, the Hybrid SFNet model was retrained using the class-weighted binary cross-entropy loss described earlier. The baseline model refers to the original configuration trained using standard (non-weighted) binary cross-entropy loss, where both classes were treated equally. Table 9 presents the comparative performance between the baseline BCE model and the weighted-loss model.

Weighted loss improves fracture recall from 77% to 85%, implying a significant reduction in false negatives. More precisely, it reduces the incidence of missed fractures from 145 to 94 (about 35% decrease). Despite a minimal reduction in precision and overall accuracy, this tradeoff is acceptable clinically in emergency radiology, where a sensitivity to fracture is stressed over marginal gains in overall accuracy.

### 4.2. Ablation Studies

To better understand the contribution of individual components in the Hybrid SFNet model, ablation studies were conducted. These experiments involved systematically removing or altering specific components of the model while keeping all other factors constant. The following configurations were tested:

- Full Hybrid SFNet Model: The complete model with all components integrated, including semantic flow mechanisms, Grad-CAM for interpretability, and edge detection preprocessing.
- No semantic flow: The semantic flow mechanism was removed, and the model relied only on convolutional layers for feature extraction.
- No Grad-CAM: The Grad-CAM module for interpretability was excluded, and the model’s outputs were evaluated without visualization capabilities.
- No edge detection: The preprocessing step involving Canny edge detection was skipped, and the model was trained on raw grayscale images.

Table 10 summarizes the performance metrics for each configuration. The results demonstrate that removing any of the components negatively affects performance, particularly recall and F1-score for fracture detection.

The ablation studies confirm the importance of each component:

- Semantic flow mechanism: Removing this component reduced feature localization accuracy, which corresponded to reduced recall for fracture sites.
- Grad-CAM: Exclusion of this component maintained accuracy but reduced model interpretability, which is critical for clinical applications.
- Edge detection preprocessing: Skipping this step led to the largest performance drop, highlighting its role in enhancing model robustness through improved boundary detection.

The ablation results demonstrate that all the components have a net positive contribution to performance. Edge detection removal results in the largest performance degradation (−8% accuracy), indicating it resulted in an 8% reduction in accuracy when removed. In addition, the semantic flow mechanism enhances feature localization (+4.5% accuracy higher than baseline). Class-weighted loss additionally enhances fracture recall, raising it from 77% to 85%, leading to reduced false negatives from 145 to 94 cases (35% reduction). These results show that the performance improvement comes from the synergistic combination of architectural and optimization components.

### 4.3. False-Negative Error Analysis and Clinical Implications

Based on a qualitative radiology-based examination of 145 false negative cases, the majority of missed fractures were characterized by subtle or hairline cortical disruptions, non-displaced fractures that exhibited no apparent angulation or step-off, and fractures in the shadow of adjacent anatomical structures (i.e., ribs or carpal bones). The contrast fractures were further diminished by low-contrast fracture lucencies that co-occurred with typical trabecular patterns and fracture lines oriented parallel to the beam path. Grad-CAM indicated that the model tended to concentrate on neighboring high-contrast cortical sites but not the subtle fracture line, demonstrating that it was difficult to detect low-saliency patterns. These results are consistent with well-established diagnostic challenges in clinical radiology and show areas for improvement that include contrast-sensitive feature augmentation and segmentation-guided attention mechanisms.

False negatives are of a higher clinical significance in fracture detection work than false positives. Whereas false positives may be followed by additional imaging, follow-up examinations, or specialist consultation, false negatives may lead to missed fractures, delayed diagnosis, lack of appropriate management, and increased risk of complications, including non-union, malunion, or increased patient morbidity. Due to the impact that prompt and correct confirmation of fracture type can have on treatment choices in trauma radiology, accuracy is often weighted less positively than sensitivity. Therefore, a false negative rate like this (145 out of 626 fractured cases) in the present study demonstrates the optimization needed to improve clinical reliability and safety.

A number of methodological approaches might be available to decrease this restriction. Class-weighted or cost-sensitive loss functions can be applied to penalize false negatives more heavily during training to make fracture detection a priority as an objective. Likewise, focal loss could facilitate the detection of subtle or low-contrast fracture patterns by emphasizing hard-to-classify samples. Adjusting the decision threshold toward higher sensitivity is another feasible practice, but calibration is necessary to balance recall and precision according to clinical needs. Ensemble methods might decrease the variance in the model’s predictions and increase robustness across heterogeneous fracture presentations.

The proposed framework is now based solely on imaging data. However, the inclusion of structured clinical metadata—patient age, trauma mechanism, and radiographic projection—could lead to more context-sensitive detection of fractures and increase diagnostic performance. As in the current study, multimodal integration was not possible due to a lack of uniform and comprehensive metadata representation found in the publicly available datasets employed. For such reasons, to further facilitate model generalizability and clinical applicability, multimodal learning approaches of imaging features and structured clinical information are recommended for future investigation.

Notably, the Hybrid SFNet is developed as a clinical decision-support tool for radiologists rather than to replace expert judgment. Enhancing sensitivity to fracture is a key target for further development of the system in order to provide improved alignment with safety standards and real-world diagnostic requirements. The results of this study indicate that imbalance-aware optimization may increase fracture recall from 77% to 85% (8% absolute improvement). Adding κ and MCC guarantees that the evaluation reflects not only overall correctness but agreement beyond chance and balanced predictive capability. This becomes particularly relevant in fracture detection, where reducing false negatives is clinically more critical than maximizing overall accuracy. Using these additional metrics, the proposed Hybrid SFNet framework is tested within a more rigorous and clinically meaningful performance framework.

In addition to the quantitative evaluation, all misclassified instances (false negatives and false positives) were qualitatively described to investigate anatomical and imaging-induced error. The false-negative cases were classified mostly with minor and/or non-displaced or low-contrasting fracture lines, often covered by overlapped anatomical structures or trabecular bone structure. In a few cases, the Grad-CAM activation maps showed the proximity of cortical edges rather than the weak fracture lucency itself. False-positive cases were often attributed to projection artifacts, normal anatomical shapes, or high-contrast trabecular intersections that resemble fracture lines visually. The activation patterns indicate that the model may sometimes confuse the underlying structural complexity for pathological disruption. As representative misclassified images are not included (due to space restrictions), this qualitative analysis offers clinically useful details about the model’s performance and points to areas for further improvement.

Moreover, the approach of generative modeling, like Generative Adversarial Networks (GANs) or diffusion-based synthetic data generation, could be considered to generate realistic fracture patterns, specifically underrepresented and subtle fracture types. Furthermore, synthetic augmentation strategies can address the residual class imbalance and enhance sensitivity in terms of low contrast and rare fracture presentations.

### 4.4. Model Comparison and Explainability

The comparison of various models for single-site and multi-site fracture detection is summarized in Table 11. This highlights the performance of the proposed Hybrid SFNet model against existing approaches.

While Table 11 shows that some studies with higher classification accuracy report better average accuracy, direct comparison should be applied with caution, considering the heterogeneity of datasets, methods of preprocessing, class distributions, and evaluation methodologies used. In existing work, small or homogeneous data was used, transfer learning on large pretrained models was performed extensively, and internal validation was used for performance evaluation only. On the other hand, the current study focuses on robustness and generalization through the use of a balanced dataset, standardized preprocessing, and consistent evaluation metrics. Moreover, integrating structured feature extraction with Grad-CAM-based interpretability analysis offers clinically meaningful insights and not just fine-tuned accuracy. Therefore, although some models may report slightly higher numerical accuracy, the current study differs from prior studies by incorporating standardized preprocessing, class-weighted optimization, and Grad-CAM visualization.

**Figure 7:**
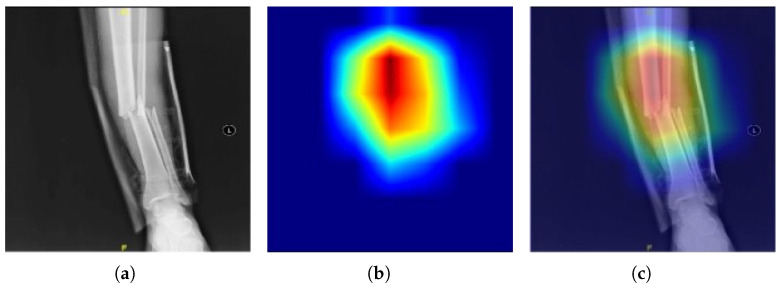
Application of GRAD-CAM on X-ray images. (**a**) Original X-ray image. (**b**) Heatmap showing regions influencing the model’s decision. (**c**) Overlay of the heatmap on the X-ray image for better visualization.

Figure 7 illustrates the application of GRAD-CAM on X-ray images for both fractured and non-fractured bones. The heatmaps generated by Grad-CAM highlight regions influencing model decisions, offering insights into the interpretability of predictions.

In addition, although the proposed model demonstrates strong performance at the image level, it was evaluated on an image-by-image basis because of the structure of the publicly available datasets, which do not contain consistent patient-level identifiers. Diagnosis decisions are made at the patient (case) level rather than per image in a clinical setting, especially in emergency radiology. Therefore, future studies need to perform a robust validation of the model on patient-level datasets to better assess its real-world clinical applicability and workflow integration.

A second limitation of this study is the absence of independent external validation. While using publicly available datasets of anatomical diversity in order to do the work, validation for the model was only done on them. This means that external validation with data from other institutions, various imaging devices, and patient populations is vital in order to assess model generalizability and robustness. Future studies should examine multi-center external validation to enhance the clinical utility of the proposed framework.

Another limitation of this study is the lack of a direct comparison of the proposed Hybrid SFNet model with radiologist performance via a structured reader study. While the model was tested versus other previously published deep learning approaches, it did not undergo a controlled human-reader benchmark on the same dataset. Such a comparison would shed light on the relative strengths, weaknesses, and complementary roles of AI systems and clinicians in fracture detection. Future work will involve prospective multi-reader studies with radiologists of varying levels of experience to gauge diagnostic performance, workflow integration, and the potential clinical impact of AI-assisted interpretation.

## 5. Conclusions

This study introduced a Hybrid SFNet model for multi-site bone fracture detection using publicly available radiographic datasets. The model integrates multi-scale feature extraction, semantic flow mechanisms, edge-enhanced preprocessing, and Grad-CAM–based visualization within a unified classification framework.

On the independent test set, the model achieved 90% overall accuracy. For fractured cases, precision was 94%, and recall was 77% under standard binary cross-entropy loss. Applying class-weighted optimization increased fracture recall to 85%, reducing false negatives by approximately 35%, while maintaining comparable overall accuracy (89%). The model achieved a Cohen’s Kappa of 0.79 and an MCC of 0.76, indicating substantial agreement beyond chance and balanced classification performance across classes.

The results show that imbalance-aware optimization improves fracture sensitivity, which is particularly relevant in clinical scenarios where missed fractures may affect patient management. Grad-CAM visualizations provided localization cues corresponding to fracture regions, supporting the interpretability of model decisions.

Limitations include the absence of external multi-center validation, patient-level evaluation, and structured comparison with radiologist performance. Future work focuses on multimodal integration with clinical metadata, supervised segmentation-classification multi-task learning, threshold calibration for sensitivity optimization, and prospective reader studies.

Overall, the Hybrid SFNet framework provides a quantitatively evaluated and interpretable approach for multi-site fracture detection in radiographic imaging.

## Figures and Tables

**Table 1:** Studies on Convolutional Neural Networks (CNNs) and transfer learning for bone fracture detection. Newly added 2025–2026 studies are highlighted in red.

Author	Dataset	Methodology	Results/Notes
Sinthura et al. [4]	Private, X-ray	Discrete Wavelet Transform (DWT) for edge detection, ANN Clustering: SFCM (Spatial Fuzzy C-Means)	Accuracy: 78%. Future work: Handle noise from X-rays.
Wang et al. [5]	Private, CT (686 mandibular fractures)	U-Net for segmentation, ResNet-50 for fracture classification	U-Net Dice: 0.943, Avg AUC: 0.965, Best accuracy: 98.28%.
Kim et al. [6]	Private, X-ray (1389 wrist)	Inception v3 with transfer learning	AUC: 0.954, Sens: 0.9, Spec: 0.88. Overfitting observed.
Ma et al. [7]	Private, X-rays	Faster R-CNN + CrackNet + Schmid filters	Accuracy: 90.11%, F1: 90.14%.
Lindsey et al. [8]	Private, Wrist X-rays	CNN with U-Net extension	AUC: 0.967.
Guy et al. [9]	Private, Pelvic X-rays	AlexNet, GoogLeNet	GoogLeNet AUC: 0.98.
Rajpurkar et al. [10]	Public, MURA	DenseNet-169	AUROC: 0.929.
Baig et al. [11]	Public, Kaggle	AlexNet transfer learning	Accuracy: 98.43%, F1: 0.9823.
Amodeo et al. [12]	Private, CT (208 patients)	ResNet-50 fine-tuned	Accuracy: 81%, AUC: 0.82.
Haitaamar et al. [13]	Public, Rib X-rays	U-Net segmentation	Accuracy: 95%.
Nguyen et al. [14]	Public, Arm X-rays	YOLOv4 detection	Accuracy: 81.91%.
Abbas et al. [15]	Public, Tibia–fibula	Faster R-CNN	Accuracy: 97%.
Sasidhar et al. [16]	Public, Humerus X-rays	VGG19, DenseNet TL	Accuracy: 92%.
Aldhyani et al. [17]	Private, X-ray	Deep CNN-based fracture diagnosis framework	Demonstrated effective automated fracture classification in clinical radiographs.
Alwzwazy et al. [18]	Private, X-ray	FracNet: End-to-end CNN architecture for fracture detection	Improved feature extraction with streamlined inference pipeline.
Yu et al. [19]	Private, X-ray	Deep learning + bone morphometric parameters	Early prediction of fracture non-union using integrated imaging features.
Belali et al. [20]	Private, X-ray	CNN-based osteoporosis fracture detection	Improved sensitivity for subtle fracture patterns.
Salman and Abu-Naser [21]	Private, X-ray	MobileNetV2 vs. ResNet50 comparison	Highlighted backbone impact on diagnostic performance.
Bhat et al. [22]	Hand radiographs	Deep + traditional ML models	Exploratory comparative analysis of fracture identification methods.

**Table 2:** Summary of studies on object detection models and image segmentation for bone fracture detection.

Author	Dataset	Methodology	Results/Notes
Moon et al. [23]	Private	YOLOX-S model trained using IoU Loss with Mixup data augmentation	Average precision: 69.8%.
Hardalacc et al. [24]	Private, 542 wrist X-rays (569 fractures)	Faster R-CNN, RetinaNet, and ensemble models (WFD-C best)	WFD-C AP50: 0.8639, AR: 0.33, Optimal LRP: 0.77. No healthy controls included.
Shan et al. [25]	Private, CT	YOLOv3 for object detection, modified attention U-Net for segmentation	YOLOv3 Precision: 0.894, Recall: 0.587, Accuracy: 83.83%. Modified U-Net Accuracy: 88.26%.
Hrvzic et al. [26]	Public, GRAZPEDWRI-DX dataset (20,327 wrist X-rays)	Modified U-Net and YOLOv4 (512 Anchors best)	Precision: 0.90, Recall: 0.89, F1: 0.89, Accuracy: 0.89.
Ju et al. [27]	Public, GRAZPEDWRI-DX dataset (20,327 wrist X-rays)	YOLOv8 with Backbone, Neck, and Head model	mAP50: 0.638. Detects nine trauma classes.
Wang et al. [28]	Public, CT (thigh bones)	ParallelNet with multiple backbones	Accuracy: 87.8%.
Wang et al. [29]	Public, Multi-site fractures	DCNN with attention mechanisms	Accuracy: 88.7%.
Hrvzic et al. [30]	Private, X-ray (children’s ulna and radius bones)	Local Shannon entropy for pixel-level segmentation, PCA for edge detection	Accuracy: 91.16%, Precision: 86.22%, Recall: 77.52%, F1: 81.64%, Specificity: 95.79%.

**Table 3:** Summary of the FracAtlas and DIDS datasets.

Dataset	Total Images	Fractured	Non-Fractured	Fracture Types	Resolution
FracAtlas	4900	2445	2455	Wrist, femur, rib, etc.	512 × 512 px
DIDS	4083	717	3366	Not specified	1024 × 1024 px

**Table 4:** The distribution of the combined dataset after merging two original datasets.

Set	Fractured	Non-Fractured	Total
Training Set (64%)	2024	3724	5748
Validation Set (16%)	506	931	1437
Test Set (20%)	632	1165	1797
Total	3162	5821	8983

**Table 5:** Dataset distribution before and after augmentation for each class.

	Fractured	Non-Fractured	Total
Before Augmentation	2007	3741	5748
Augmented Images	40,000	74,559	114,559
After Augmentation	42,007	78,300	120,307

**Table 6:** Layer-wise architecture of the proposed SFNet model.

Layer	Type	Kernel/Size	Stride	Output Shape	Parameters
Input	Input Layer	–	–	224×224×3	0
Conv1	Conv2D (32 filters)	3×3	1	224×224×32	896
BN1	BatchNorm	–	–	224×224×32	128
MaxPool1	MaxPooling	2×2	2	112×112×32	0
Conv2	Conv2D (64 filters)	3×3	1	112×112×64	18,496
BN2	BatchNorm	–	–	112×112×64	256
MaxPool2	MaxPooling	2×2	2	56×56×64	0
Conv3	Conv2D (128 filters)	3×3	1	56×56×128	73,856
BN3	BatchNorm	–	–	56×56×128	512
MaxPool3	MaxPooling	2×2	2	28×28×128	0
GlobalAvgPool	Global Average Pooling	–	–	128	0
Dense1	Fully Connected (ReLU)	–	–	128	16,512
Dropout	Dropout (0.5)	–	–	128	0
Output	Dense (Sigmoid)	–	–	1	129
Total Trainable Parameters					110,785

**Table 7:** Training hyperparameters used for the proposed SFNet model.

Hyperparameter	Value
Optimizer	Adam
Initial Learning Rate	$1\times 10^{-4}$
Batch Size	32
Number of Epochs	10
Loss Function	Binary Cross-Entropy
Early Stopping Patience	5 epochs
Dropout Rate	0.5
Input Image Size	224×224
Weight Initialization	He Normal
Learning Rate Scheduler	ReduceLROnPlateau (factor = 0.1)
Data Augmentation	Rotation (±15°), Horizontal Flip, Zoom (0.1)

**Table 8:** Result of Hybrid SFNet Model.

	Precision	Recall	F1-Score
Non-fractured	0.89	0.98	0.93
Fractured	0.94	0.77	0.85
Accuracy	0.90		
Cohen’s Kappa (κ)	0.78		
MCC	0.74		

**Table 9:** Performance comparison between baseline BCE and class-weighted BCE.

Model	Accuracy	Precision (Fractured)	Recall (Fractured)	F1-Score (Fractured)	Cohen’s Kappa (κ)	MCC
Baseline BCE	0.90	0.94	0.77	0.85	0.78	0.74
Weighted BCE	0.89	0.88	0.85	0.86	0.79	0.76

**Table 10:** Ablation study showing the contribution of each component in the Hybrid SFNet framework.

Configuration	Edge	Semantic Flow	Weighted Loss	Accuracy (%)	Recall (%)	F1-Score (%)	Cohen’s Kappa (κ)	MCC
Baseline CNN	No	No	No	82.0	75.0	78.0	0.78	0.74
+Semantic Flow	No	Yes	No	83.5	78.0	80.0	0.79	0.75
+Edge Detection	Yes	Yes	No	90.0	87.0	90.0	0.80	0.78
+Weighted Loss	Yes	Yes	Yes	89.0	85.0	86.0	0.83	0.81

**Table 11:** Comparison of models for single-site and multi-site fracture detection.

Ref.	Scope	Model	Accuracy
Haitaamar et al. [13]	Single site fractures	U-Net	95%
Nguyen et al. [14]		YOLOv4	81.91%
Ma et al. [7]		Faster R-CNN	90.11%
Abbas et al. [15]		Faster R-CNN	97%
Sasidhar et al. [16]		VGG19, DenseNet121, DenseNet169	92%
Wang et al. [28]	Multi-site fractures	Two-stage R-CNN	87.8%
Wang et al. [29]		DCNN	88.7%
Proposed Model		Hybrid SFNet	90%

## Data Availability

The dataset with code implementation is available at https://www.kaggle.com/code/wijdanalmutairi/bone-frac-combined-dataset (accessed on 1 January 2026).
